# Imaging biomarkers in the idiopathic inflammatory myopathies

**DOI:** 10.3389/fneur.2023.1146015

**Published:** 2023-04-25

**Authors:** Adeel S. Zubair, Sharfaraz Salam, Mazen M. Dimachkie, Pedro M. Machado, Bhaskar Roy

**Affiliations:** ^1^Division of Neuromuscular Diseases, Department of Neurology, Yale University School of Medicine, New Haven, CT, United States; ^2^Department of Neuromuscular Diseases, UCL Queen Square Institute of Neurology, University College London, London, United Kingdom; ^3^Department of Neurology, The University of Kansas Medical Center, Kansas City, KS, United States; ^4^Centre for Rheumatology, Division of Medicine, University College London, London, United Kingdom

**Keywords:** myositis—diagnosis, imaging, MRI, inclusion body myositis (IBM), electrical impedance myography, muscle ultrasound

## Abstract

Idiopathic inflammatory myopathies (IIMs) are a group of acquired muscle diseases with muscle inflammation, weakness, and other extra-muscular manifestations. IIMs can significantly impact the quality of life, and management of IIMs often requires a multi-disciplinary approach. Imaging biomarkers have become an integral part of the management of IIMs. Magnetic resonance imaging (MRI), muscle ultrasound, electrical impedance myography (EIM), and positron emission tomography (PET) are the most widely used imaging technologies in IIMs. They can help make the diagnosis and assess the burden of muscle damage and treatment response. MRI is the most widely used imaging biomarker of IIMs and can assess a large volume of muscle tissue but is limited by availability and cost. Muscle ultrasound and EIM are easy to administer and can even be performed in the clinical setting, but they need further validation. These technologies may complement muscle strength testing and laboratory studies and provide an objective assessment of muscle health in IIMs. Furthermore, this is a rapidly progressing field, and new advances are going to equip care providers with a better objective assessment of IIMS and eventually improve patient management. This review discusses the current state and future direction of imaging biomarkers in IIMs.

## Introduction

Idiopathic inflammatory myopathies (IIMs) are a heterogeneous group of acquired muscle diseases characterized by muscle inflammation, weakness, and other extra-muscular manifestations (1–4). Classically, the IIMs were sub-grouped into dermatomyositis (DM), polymyositis (PM), and inclusion body myositis (IBM) (5). Increasing evidence suggesting that myositis-specific antibodies (MSA) can help define subgroups of patients with different phenotypes, prognosis, and response to treatment has favored the development of a new classification system that groups IIMs into DM, IBM, immune-mediated necrotizing myopathy (IMNM), and antisynthetase syndrome (ASS) (6). All IIMs, except for most IBM cases, usually present with acute or subacute symmetric proximal weakness. IBM typically presents with insidious onset asymmetric quadriceps muscle weakness with frequent long finger flexors involvement; patients often develop dysphagia (7, 8). Patients with DM have characteristic skin changes such as periorbital edema and erythema (heliotrope rash), and Gottron papules on the dorsum of hands and fingers. The later rash overlaps can also be seen in the ASS.

Disease severity of IIMs can vary, and patient quality of life can be significantly impaired (1, 2). The management of IIMs is challenging and requires a multi-disciplinary team including rheumatologists, neuromuscular specialists, dermatologists, physical, occupational, and speech therapists, pulmonologists, and cardiologists. The role of imaging technologies in the diagnosis, assessing disease activity and treatment response, and monitoring disease progression of the IIMs is increasingly being recognized. The objective nature of these technologies can complement the available outcome measures and will facilitate future clinical trials. In this study, we review the application of imaging technologies as an objective biomarker of IIMs, their limitations, and future directions.

## Methods

A PubMed search was done for the terms “myositis and biomarkers,” “MRI and myositis,” “electrical impedance myography and myositis,” and “ultrasound and myositis” for all English language literature until 14 February 2023. Overall, this resulted in a total of 2,672 results which were then screened by AZ for applicability to this topic. This resulted in 199 publications that were reviewed for this study (Supplementary Figure 1).

## Magnetic resonance imaging

**Figure 1:**
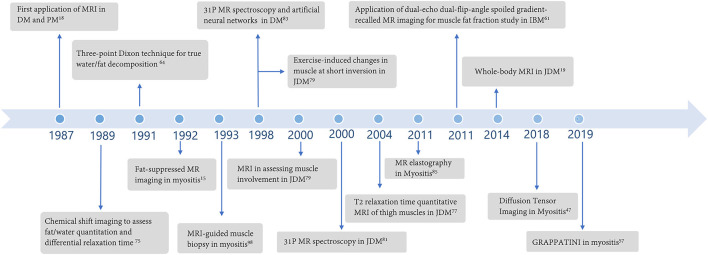
Major developments regarding the application of magnetic resonance imaging in idiopathic inflammatory myopathies over the years.

**Figure 2:**
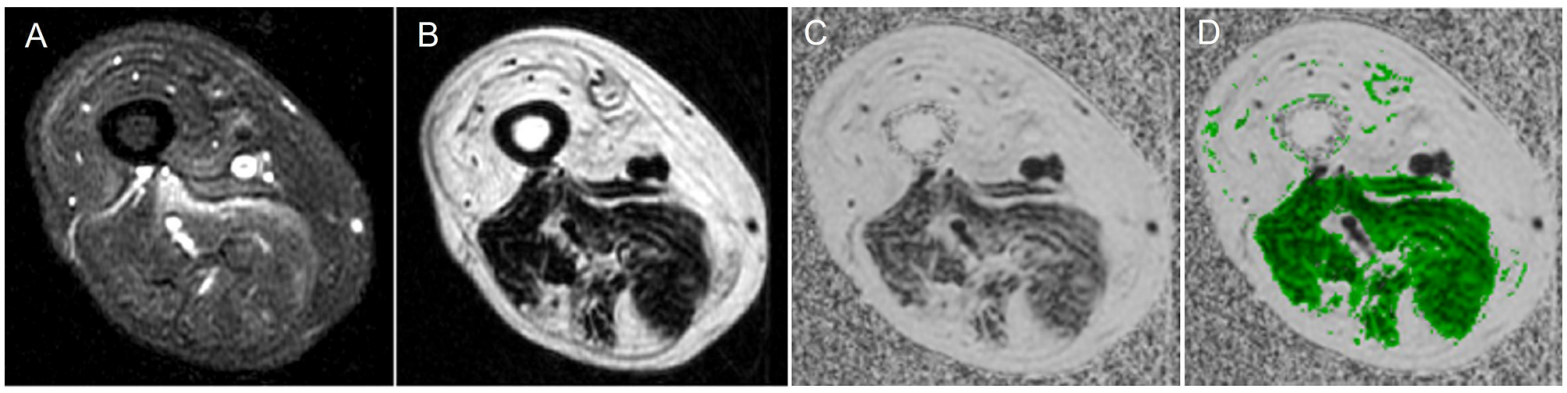
Magnetic resonance imaging of muscle showing water vs. fat image and a fat fraction map. **(A)** Water image, **(B)** fat image, **(C)** fat fraction map, and **(D)** fat fraction map with muscle region overlaid.

Magnetic resonance imaging (MRI) has been used to assess muscle involvement in myositis for over three decades (Figure 1), and it is the imaging modality of choice for the evaluation and follow-up of muscle changes in myositis patients. MRI can distinguish between active muscle inflammation vs. chronic muscle damage and is a tool to optimize sample selection for muscle biopsies (Figure 2) (9, 10). Additionally, MRI patterns of muscle involvement can help distinguish between IIMs such as DM/PM and IBM.

### MRI characteristics of normal muscles

Normal, healthy muscle generates an intermediate signal intensity, slightly higher than water and much lower than fat using T1 weighted image sequences. On the contrary, T2-weighted sequences generate a much lower signal for healthy muscle in comparison to fat and water (11, 12). With short-tau inversion recovery sequences (STIR) or fat-suppressed T2-weighted sequences, normal muscle signal intensity is lower than the signal intensity of pure/free water molecules but higher than pure/free fat molecules (12–15). Usually, fat replacement is better captured by T1-weighted images, while muscle edema is better detected with STIR or fat-suppressed T2 images.

MRI captures a wide area of muscle and provides more detailed information than computed tomography and muscle ultrasound. Moreover, it can identify changes in the deep muscles which can be particularly challenging with muscle ultrasound and electromyography. Thus, MRI is particularly helpful for the identification of muscle involvement patterns that can be used to distinguish between IIMs and myopathies in general.

### MRI changes in IIMs

In myositis, there is muscle edema in the early stage of active muscle disease and muscle atrophy and fatty replacement in the later stages of muscle damage which alters the normal MRI signal of muscles. However, in some cases, both can co-exist (16, 17). The initial study by Kaufman et al. (18) showed higher T1 signal intensity, atrophy, and fat replacement in active DM and PM. Edema is noted in the early stage of myositis even in the absence of clinical weakness and with normal creatine kinase (CK) (18, 19). MRI is ~80%−90% sensitive in showing muscle edema in active myositis (13, 20). Studies from juvenile DM showed that 76%−97% of patients will have muscle edema on MRI (21, 22). A study showed that 56% of patients with active DM, and 15% of patients with PM can have muscle edema on MRI even without any elevation of CK level. The pattern of muscle edema in DM and PM can vary; edema is usually diffuse in PM and patchy and ill-defined in DM. Moreover, edema can spread to subcutaneous tissue and fascia in DM (19, 23).

The pattern of muscle involvement can be helpful to distinguish between IIMs (24–27). In PM, inflammatory changes, reflected as edema, are symmetric and affect proximal upper and lower extremity muscles. Usually, the adductor group of muscles is more involved in PM, whereas DM frequently involves the quadriceps (9, 28, 29). On the contrary, MRI findings in sporadic IBM patients are more extensive in the lower extremities and are usually asymmetric; however, inflammatory changes are seen in all affected muscles and prevalent at all stages of the disease (16, 28, 30–32). Typically, findings are more severe in distal muscles, and fat infiltration is a frequent pathologic finding in patients with IBM (31). The anterior compartment of thigh muscles is usually more affected, but the rectus femoris is relatively spared (16, 31). Relative sparing of pelvic muscles can be seen. Among the distal muscles, the medial gastrocnemius is usually the most affected (16, 31). The fat infiltration in the individual muscles of these patients is heterogeneous in terms of the proximal-to-distal gradient, and the severity of fat infiltration correlated with worse clinical scores (32). Edema and atrophy have been reported to be present together in both PM and IBM (28). Undulating fascia sign is the presence of wavy fascia between the severe atrophic and fat-infiltrated vastus muscles commonly seen in IBM. Undulating fascia sign is associated with more severe disease and poor clinical outcomes. However, this sign is not specific to IBM and can be seen in conditions with severe atrophy and fat infiltration of quadriceps including advanced PM (28, 31).

Patients with IMNM often have severe lower limb muscle edema with fatty replacement and atrophy, and pelvic muscles and adductors are usually more affected than patients with DM (33). Patients with ASS often have significant subcutaneous tissue edema and relative sparing of adductor muscle, which is similar to DM but distinct from IMNM (24). Despite some similar findings with DM, patients with ASS may have less symmetric involvement and more common myofascial edema of tensor fascial lata (24).

Apart from the standard T1 and T2 (or STIR), other MRI sequences also can provide valuable information on muscle health in myositis. Diffusion-weighted imaging (DWI) measures the mobility of free water in the living tissue; areas of increased free water content result in increased diffusivity, and apparent diffusion coefficient (ADC), an index of diffusivity, is higher in these regions and can identify muscle edema that is comparable to STIR (34). Apparent diffusion coefficient (ADC) values in patients with myositis increased in affected muscles compared to that of normal muscles (35–38).

Muscle edema and fat deposition are not unique to IIM and can be seen in denervation changes (including disorders of the motor neurons), neoplasm, infection, and muscle injury (20, 39–44). Differentiating neurogenic changes from IIMs can sometimes be difficult. The overall pattern of muscle involvement and comparing it to “classic” forms of IIMs can be helpful (42). Muscle MRI can be useful as a clinical tool to identify a pattern (with a sensitivity to detect selective patterns in the rigidity of the spine in relation to the genetic diagnosis reported as 0.9) (45). Furthermore, the identification of muscle “islands” or small areas of muscle tissue with normal signal intensity surrounded by areas with intensity similar to subcutaneous fat on imaging is usually associated with neurogenic etiology (42, 46).

### Other MRI sequences for evaluation of IIMs

Diffusion tensor imaging (DTI) evaluates the anisotropic diffusion of water molecules which can help characterize physiological and microstructural properties of skeletal muscles and architectural organization (47–49). DM patients have lower pseudo-diffusion and volume in quadriceps muscles. Moreover, static dynamic diffusion imaging metrics correlated with T1/T2 scores (50). However, in another study, the mean apparent diffusion coefficient was higher in patients with DM, but no significant difference in fractional anisotrophy was noted between edematous and normal muscles. Traditional DTI has limitations including a reduced signal-to-noise ratio (SNR) on DTI images and prolonged study time (47–49). Discrepancies in DTI measures can be due to many issues, potentially including unsatisfactory fat suppression. Simultaneous multi-slice (SMS) accelerated echo planar imaging (EPI) DTI which combines simultaneous excitation of multiple slices during acquisition and spatially encodes their signals in a simultaneous manner can capture fast images and shows lower fractional anisotrophy (47, 51).

The problem of long scanning times is not limited to DTI imaging but also applies to traditional MRI T2 sequences which can take up to 30 min per thigh (52, 53). A new technique based on a multi-echo spin-echo sequence including a reconstruction method that combines model-based accelerated relaxometry by iterative non-linear inversion (MARTINI) with generalized auto-calibrating partial parallel acquisition (GRAPPA) can acquire much faster images (54–56). GRAPPATINI utilizes the characteristics of the two methods and can significantly shorten the acquisition time of T2 mapping (52, 57). However, elevated GRAPPATINI-generated T2 values were seen in some non-edematous muscles which were normal in conventional MRI DM patients (52).

### Quantitative MRI

Qualitative assessment of muscles can identify a specific pattern and can help in diagnosis but is subjected to individual biases and may not accurately represent disease progression and treatment response. There has been an unmet need for a more objective measure of muscle damage in IIMs (58, 59).

Semi-quantitative methods use visual assessment by an evaluator who assigns a numerical grade on an ordinal scale. The Mercuri scale, a commonly used semi-quantitative grading score scale, grades muscle images between 0 and 4 (0 being normal muscle and four representing end-stage muscles with severe damage) and was created to mitigate this problem to some extent (60–62). This scale helped in standardizing muscle imaging and is relatively easy to administer, but it is essentially an ordinal scale. Ordinal scales may lack sensitivity and can have subjective bias. A study by Kubinova et al. reported a large variability among different scoring approaches for muscle MRI (12, 63).

Quantitative MRI has the potential to be a useful biomarker in clinical practice and in the context of clinical trials (58). They always use continuous scales to measure muscle changes more precisely. Fat fraction analysis, transverse relaxation time (T2), and magnetization transfer ratio are the commonly used quantitative muscle MRI techniques which is expected to be more accurate than the commonly used manual ordinal grading system (59).

Computer-based analysis of pixel intensity values is a commonly employed quantitative muscle MRI technique (59). Quantitative MRI techniques can determine the functioning muscle area or remaining muscle area (RMA) (16). RMA can be estimated by using the cross-sectional area. A decrease in RMA has been shown to be associated with reduced knee extension strength on myometry in IBM (16).

**Figure 3:**
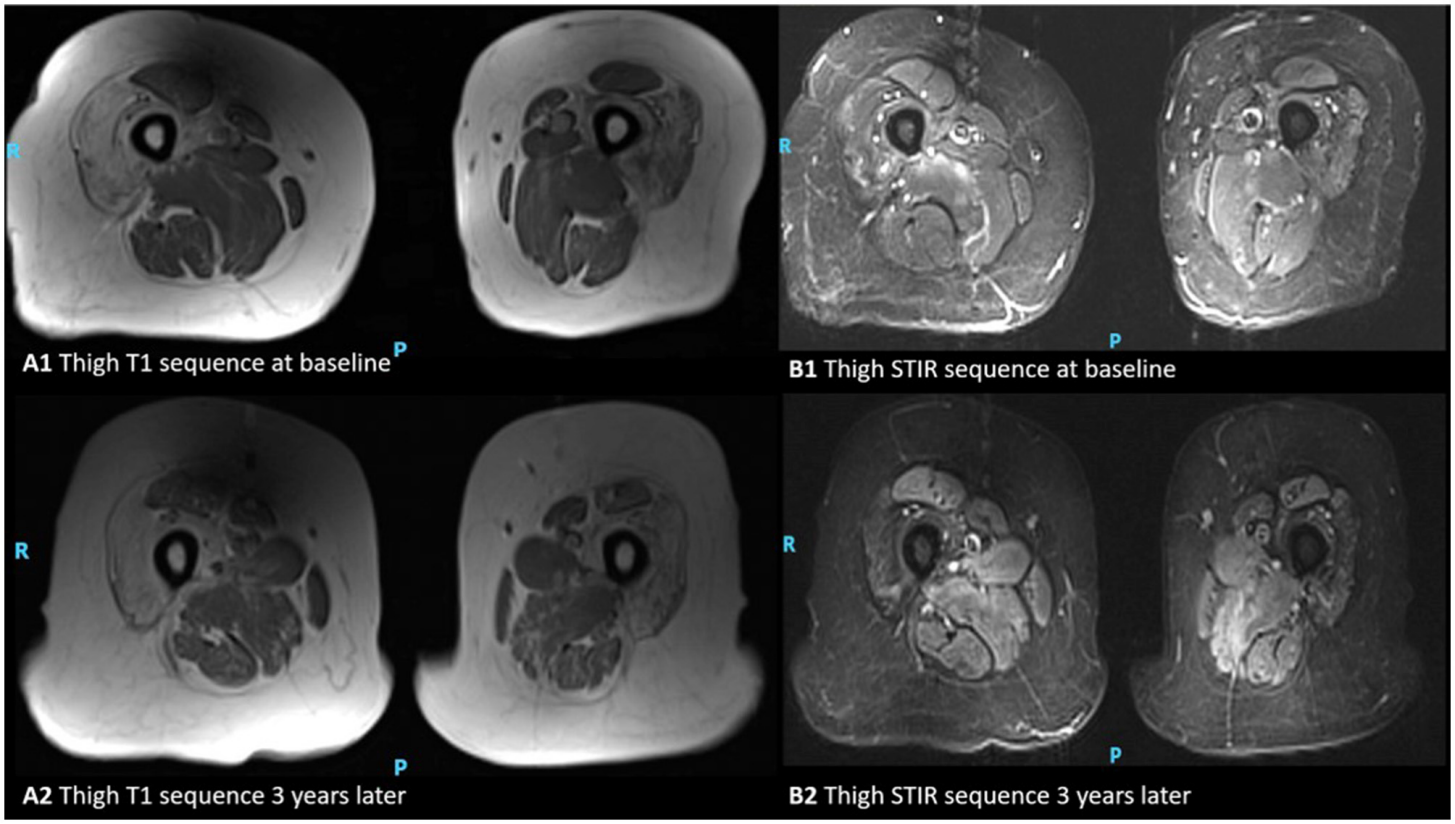
MRI appearances of thigh muscles in a patient with inclusion body myositis. Axial T1-weighted images of the thigh at the baseline and 3 years later **(A1, A2)**, and axial STIR respectively **(B1, B2)**. This figure illustrates progressive intramuscular fat accumulation with initial sparing of the rectus femoris as hyperintensities on T1-weighted sequence and acute muscle inflammation is evident as hyperintensity on STIR images (P, posterior; R, right).

A 3-Point-Dixon MRI fat-water imaging quantifies tissue fat content on a 0%−100% fat-fraction (FF) scale and has been previously used in neuromuscular diseases (Figure 3) (64–67). In Duchenne muscular dystrophy, Dixon MRI has been shown to be more precise and reliable than visual radiological methods (68). If optimally performed, the magnetization transfer ratio (MTR) excludes lipid contribution, and it is similar to water T2 relaxation time; in that, it also is sensitive to changes in the water distribution (69). In IBM, T2 and MTR showed early changes in muscles before significant intramuscular fat accumulation, providing potential measures of early disease before irreversible changes occurred (70–72). Furthermore, the whole muscle fat fraction increased at the calf level and thigh level in patients with IBM over 1 year, and the changes in fat fraction were more consistent compared to changes in longitudinal T2 and MTR changes (16). In particular, increased lower limb FF was shown to have a negative association with the Medical Research Council Sum score, lower limb components of IBM Functional rating scale (IBM-FRS) score, Rankin score, and 36-Item Short Form Health Survey questionnaire in IBM patients (16, 31, 32).

T2 relaxation time has been referenced as a more objective marker to assess muscle disease. T2 relaxation is sensitive to changes in water distribution and lipid content, and it is an objective marker of muscle health. However, fat replacement in advanced IIM can confound the interpretation of muscle T2 values and published T2 values for fat vary significantly (73–76). Combination of MRI estimation of fat fraction with a bi-exponential T2 modeling procedure can result in fat-corrected T2 (fc-T2) maps. Yao et al. published a study showing that application of fc-T2, fat fraction (FF), and muscle T2 to MRI evaluation of IIM disease activity can improve study precision and is amenable to automation (73). In children with JDM, invasive procedures like EMG and muscle biopsy are less frequently used. T2 relaxation times in JDM patients can quantify areas of inflammation and correlate with other measures of disease activity (77–79).

### MRI assessing muscle metabolism

Quantification of muscle metabolism by measuring pH and high-energy phosphate metabolites [phosphocreatine, inorganic phosphate, and adenosine triphosphate (ATP)] by phosphorus-31 magnetic resonance spectroscopy (P-MRS) can identify metabolic abnormalities in DM, juvenile dermatomyositis (JDM), and also treatment response (9, 80, 81). Mean ATP and phosphocreatine values are much lower in the thigh muscles of patients with JDM (81). Post-exercise P-MRS indices are impaired in DM with prolonged post-exercise recovery likely related to impaired perfusion (80). In DM, JDM, and PM, these metabolic abnormalities improve with corticosteroid therapy (81–83). On the contrary, patients with IBM have abnormal resting metabolites but normal post-exercise recovery parameters (84). While MRS can be a useful modality that can differentiate between subtypes of IIMs and assess treatment response, it is limited by cost and availability, and still mostly used for research purposes (9, 20, 39).

### Magnetic resonance elastography

Magnetic resonance elastography (MRE) can provide an assessment of muscle stiffness based on the propagation of shear waves (85, 86). In a limited sample of patients with PM, DM, and JDM, a statistically significant reduction in MRE imagery was noted when compared to healthy controls in a relaxed state (87).

### Whole-body MRI

Whole-body MRI is useful to capture distal and patchy disease activity that was not appreciated clinically and would have been missed by dedicated regional imaging (88). It is becoming particularly popular in pediatric populations with the advent of increasingly efficient MRI scanners (19, 89). Whole-body MRI can also play an important role by potentially detecting occult malignancy in DM and determining if there is a paraneoplastic etiology (9, 90–93). This can be valuable as older DM patients with dysphagia, anti-TIF1γ and anti-NXP2 seropositivity, and cutaneous ulceration have a higher risk of developing cancer (93).

### Use of MR imaging in clinical practice

Only limited classification criteria have incorporated MRI as a variable in the classification of IIM (9). Addition of muscle MRI and myositis-specific antibodies have been shown to improve the diagnostic accuracy of the Bohan and Peter criteria as part of the updated Targoff classification (94). Notably, the Targoff criteria from 1997 allowed the incorporation of abnormal MRI in the context of normal CK (9, 95). Muscle MRI is however not part of the current European League Against Rheumatism/American College of Rheumatology (EULAR/ACR) 2017 classification criteria for myositis (4). These are the currently widely accepted myositis classification criteria. An Australian-validated study of the EULAR/ACR 2017 myositis classification criteria showed that adding MRI as a covariate would improve the probability of IIM diagnosis and should be considered as part of a future revision of these criteria (96). However, MRI has also been used to target the best muscle sample for biopsy and to help avoid missing pathological support for the diagnosis (97, 98). In patients with IMNM muscle, MRI is a sensitive biomarker for monitoring disease activity and therapy response. Patients with higher STIR changes at the baseline were more prone to fatty replacement (99). In addition to its use as a guidance tool, some have suggested that imaging should be considered as an alternative to muscle biopsy. This is not a widely accepted approach due to the lack of specificity of signal changes as indicated earlier. In a study comparing muscle biopsy findings with MRI images, it was shown that there was a statistically significant association between the inflammatory infiltrate and both muscle and fascial edema, suggesting that key MRI findings correlate with the main features of DM muscle biopsy making them a potential surrogate marker of disease activity (100). While not as specific for IIM as a muscle biopsy, MRI does have a positive predictive value equivalent to that of biopsy (97 vs. 100%) and a better negative predictive value (64 vs. 38%) (13). As MRI has been shown to be able to assess treatment response, it is intuitive that MRI may guide therapeutic decisions and can be used to determine refractory diseases. However, such a role of MRI is yet to be established and will require a comparison with the commonly employed traditional tools of disease severity scale and clinical assessments.

### Limitations of MRI

While MRI is a very useful imaging biomarker of IIM, it has some limitations (Table 1). First, prolonged supine positioning holding a steady position without any extremity movement can be difficult for some patients. Some muscles may be difficult to be quantitively imaged such as the deep finger flexors. Similarly, patients with claustrophobia find MRI unpleasant and often require pre-medication to endure the procedure. Some patients may have implanted devices that are not compatible with MRI. MRI is usually available in the developed countries, but availability can be limited in many parts of the world and the cost of MRI can be significant. Furthermore, we must consider the cumulative cost of serial MRIs to assess disease progression or treatment response in IIMs. Apart from the logistic considerations, one major limitation of MRI is the lack of a widely validated objective scale to assess the extent of muscle disease. Quantification of muscle disease by MRI is challenging due to the variable intensity of characteristic muscle signal changes. Currently, no universally accepted scoring systems for the evaluation of muscle MRI findings exist (59, 63). Newer technologies, such as the three point-Dixon method to quantify fat deposition in muscle is valuable, but such technologies are still used only for research purposes and not widely available for clinicians. Furthermore, these methods depend on specific software-based analysis, and their reproducibility from different scanners is yet to be established. There have been limited attempts of using computer analysis of MRI image pixel values to quantify the degree of disease; however, it was shown that neither the computer algorithm nor visual analysis method was able to separate moderate disease from severe disease. A standardized, validated method for quantifying MRI findings in IIM may help with efficient diagnosis, accurate interpretation of research data, and valid comparisons across studies (17, 63). Machine learning, particularly the use of artificial intelligence with deep learning technology, has shown promising results in IIMs and other muscle disorders and has the potential to address some of the limitations of MRI (101–103).

**Table 1:** Advantages and disadvantages of different imaging technologies.

	**Advantages**	**Disadvantages**
Magnetic resonance imaging	1. High resolution images 2. Objective measures; not dependent on operator 3. Captures wide area of muscle 4. Has been widely used in IIM	1. May not be widely available 2. Expensive 3. Takes a relatively longer time to complete than other modalities 4. Lack of standardized protocol for muscle imaging 5. Lack of widely accepted and easily available quantification
Ultrasound	1. Easily accessible 2. Can be performed at bedside/clinic setting 3. Rapid screening	1. Limited by the skill of the operator 2. Quality of the image can be limited by anatomy and muscle depth 3. Muscle edema may result in too small a change in echogenicity
Electrical impedance myography	1. Less resource intensive 2. Minimal post-processing required 3. Rapid screening 4. Can be performed at bedside/clinic setting	1. Still a research tool and not widely available 2. Only provides quantified values, but no images 3. The interpretation of numerical parameters is not well-established in the clinical setting

## Ultrasound

Ultrasound imaging of the muscle tissue is becoming an important tool in neuromuscular medicine given its non-invasive nature, ease of use, and improved resolution for soft tissue structures (104–107). Ultrasound was the first imaging technique available for the evaluation of muscle disease. Normal muscle fascicles appear hypoechoic and are separated by echogenic fibro-adipose or perimysial connective tissue. Connective tissue and fat replacement in muscles is seen as increased echogenicity (108–111). Quantitative muscle ultrasound by assessing the gray-scale level (GSL) and quantified back-scatter analysis (QBA) has been found to correlate with functional status and worsening disease (112, 113). Muscle ultrasound is about 80% sensitive in diagnosing IIMs with a positive predictive value of 95% (114, 115).

Different IIMs have typical but non-specific ultrasound features (116, 117). In DM/PM muscle, inflammation and edema indicating disease activity are not well-discriminated by a simple assessment of muscle echo intensity. Muscle edema can lead to low echogenicity, but the change is too small to make a definite diagnosis of edema (118). In general, acute myositis accompanies normal or slightly swollen muscle size with relatively low intensity. However, in juvenile DM, muscle echo intensity has been surprisingly reported to first increase and then normalize in 6/7 successfully treated cases, but the seventh case had echogenicity persistently increased suggesting early fibrosis (119). In chronic stages of myositis where there is fat replacement and fibrosis, higher echo-intensities and decreased muscle thickness are apparent.

IBM has been studied with quantitative muscle ultrasound. Affected muscles show an increased echogenicity on the ultrasound image which indicates the replacement of muscle with fat and fibrosis (120–123). IBM has a specific pattern of muscle involvement that can be used to help distinguish it from other diseases. Increased echogenicity within flexor digitorum profundus (FDP) and medial gastrocnemius is highly supportive of IBM (121, 124). The FDP to flexor carpi ulnaris (FCU) echogenicity contrast, a pattern of higher echo intensity in the FDP than in the FCU, can help in discriminating IBM from DM and amyotrophic lateral sclerosis. Similarly, gastrocnemius echo intensity is significantly different from soleus echo intensity in IBM when compared to DM (121, 125).

Ultrasound has also been used to assess diaphragm thickness in myositis and allows for the assessment of anatomy and function (126). Respiratory muscle involvement is reported in IIMs (127); in IBM, data are limited about prevalence and impact on functional capacity partly due to limited methods of assessing diaphragmatic involvement (128). Ultrasound has been shown to be effective in screening patients with IBM and has shown that diaphragm involvement in IBM is related to disease duration and has detrimental effects on exercise capacity and lung function (128). More recently, studies have also shown utility in the use of ultrasound to assess diaphragm involvement in patients with immune checkpoint inhibitor-related myositis (129).

Musculoskeletal power Doppler ultrasonography (PDUS) has been used to diagnose and measure vascularity in inflammatory disorders. PDUS allows simultaneous assessment of disease activity at more sites than contrast-enhanced MRI (130). A PDUS study in patients with DM and PM showed that increased blood flow was associated with angiogenesis that accompanied fasciitis in patients with DM but not PM, possibly permitting earlier diagnosis of patients with DM (130).

Shear wave elastography (SWE) is an ultrasound-based technique that has been studied in tendinopathy, muscle spasticity, and Duchenne muscular dystrophy (131–135). SWE provides a quantitative measure of tissue stiffness by taking advantage of differences in the relative hardness of soft tissues when an external force is applied to a tissue boundary. Thigh muscle stiffness as measured by SWE was shown in one study to be lower in active IIM patients as compared to healthy controls, and the reduced muscle stiffness was likely associated with muscle weakness and MRI signs of edema and atrophy (136). Recent studies have shown evidence that SWE can have utility as an imaging biomarker that can lend support to IIM diagnosis (137, 138).

Machine learning has been studied on ultrasound in patients with myositis; one study showed that the deep learning approach performed better than conventional machine learning and required no user intervention (118). Newer models are being tested which show promise in the utilization of advanced machine learning processes to help accurately make diagnoses (139).

One additional utility is the use of ultrasound to conduct guided biopsies. Ultrasound can provide images in real time as well as identify abnormal muscle morphology, making it well-suited to target muscle biopsies with improved tissue diagnostic yield. A disadvantage of using ultrasound-guided needle muscle biopsy can be under-sampling when compared to an open biopsy resulting in an inadequate amount of specimens or non-diagnostic tissues.

The are several advantages to muscle ultrasound. It can be performed quickly at the patient bedside and does not require prolonged maintenance of a posture. It is less expensive than muscle MRI. However, despite its convenience and portability, it cannot replace MRI in assessing disease activity in the IIMs at the present state. Part of the issue with ultrasound is that it is limited in its ability to identify edema in comparison to other modalities (26, 140, 141) (Table 1).

## Electrical impedance myography

Electrical impedance myography (EIM) is a non-invasive technique that uses subthreshold electrical current to measure the obstruction to current flow (142). It involves the application of a high frequency, low-intensity alternating electrical current via two surface electrodes attached to the skin, and the resultant voltage is measured using a second set of electrodes (143), and usually reactance, resistance, and the phase angle are the measured parameters (142, 144). Changes in the underlying structure and composition of the muscle caused by the progressive disease change alters these parameters (145, 146). Resistance is the measure of the difficulty of passing an electrical current through a tissue and provides a measure of water content. Fat replacement results in higher values. Reactance measures the capacitive nature of myofibers and is diminished with worsening disease severity. Finally, the phase angle (arctan of reactance/resistance) decreases with worsening disease conditions. EIM is less resource intensive than clinical testing modalities; in that, it requires minimal time and training of staff as well as no volitional effort of the patient, and there is little postprocessing required (147).

An animal study of intra-muscular EIM to assess muscle inflammation showed that changes in low-frequency EIM parameters are sensitive to the presence of inflammatory infiltrates and have the potential to serve as a simple means of quantifying the presence and extent of inflammation without the need for biopsy (147). In facioscapulohumeral muscular dystrophy (FSHD), EIM was compared with MRI Dixon and was noted to have a strong correlation with structural MRI features lending to EIM being a potentially useful biomarker in FSHD trials (142). Other human studies in an array of disease states including Duchenne muscular dystrophy, amyotrophic lateral sclerosis (ALS), and spinal muscular atrophy (SMA) have shown that localized impedance measurements over specific muscles can result in clinically valuable data (148–150). Additionally, incorporating measurements of muscle anisotropy, another EIM parameter, can improve the reproducibility of the EIM technique as well as help distinguish myopathic and neurogenic diseases (151, 152). Technological improvements have also assisted in advancing EIM including the creation of a portable system for the assessment of neuromuscular diseases. Studies have indicated that the system was able to obtain measurements of the complex impedance of the muscle tissue rapidly and accurately (143). EIM has been shown to capture muscle inflammation in patients with inflammatory myopathy. EIM phase values from thigh muscles of affected individuals were lower and correlated with muscle strength (153). EIM has also been recently studied in IBM and was shown to detect changes in the muscle health of patients and to correlate with standard functional outcome scales. To complement this cross-sectional study, longitudinal studies are needed to validate EIM as a potential biomarker of IBM.

EIM provides information on muscle health but does not provide information on patient's function and statistical significance does not always clearly translate to clinical significance. EIM has not been used as the sole primary outcome measure in clinical trials but has been used as an exploratory outcome measure in a natural history study of SMA (154, 155). EIM has some advantages over MRI including rapid acquisition and the ability to complete in an outpatient setting. Additionally, it is less expensive than MRI and can be adapted to any muscle allowing for a tailored assessment of disease progression and medication response.

## Positron emission tomography

Fluorine-18 (^18^F)-labeled fluorodeoxyglucose positron emission tomography (FDG PET) is generally used for detecting malignancies; however, FDG also accumulates in inflammatory lesions where glucose-consuming inflammatory cells infiltrate. There have been a few studies looking into the utility of FDG PET in myositis (156). A study of 24 patients with PM/DM demonstrated increased FDG uptake in proximal muscles as compared to controls (33 vs. 2%) (157). Another study looked at the maximum standardized uptake value (SUVmax) in the proximal muscles of all four limbs in patients with PM/DM and showed that the proximal muscle SUV ratio was higher in patients with PM/DM than controls (158). A follow-up study of 33 patients with PM/DM compared with 22 patients with ALS showed that visually-identified FDG (vFDG)-positive regions correlated strongly with mean SUVmax and that when compared to patients with ALS, the mean SUVmax was significantly higher in patients with PM/DM (159).

Amyloid positron emission tomography (amyloid-PET) has been investigated as a diagnostic tool for IBM and differentiates it from other IIMs such as PM (160–162). These studies have used a relatively small number of patients so far. Different amyloid tracers have been employed in these studies; Pittsburgh Compound B ([^11^C]PIB) and [^18^F] florbetapir. Lilleker et al. (160) performed the largest PET study to date (10 IBM patients and six PM patients) with [^18^F] florbetapir tracer. SUV ratios were significantly higher in all regions assessed in IBM patients compared to PM. Amyloid PET tracer uptake has not been shown to be associated with clinical severity in these studies (160, 162).

In patients with DM, FDG PET is also used to screen for malignancy (163). Thus, it can be used to monitor the disease activity while searching for any underlying malignancy. Notably, the role of PET scans in diagnosing and measuring disease activity in patients with interstitial lung disease has yet to be assessed (164).

## Discussion

Over the course of the last few years, there have been significant imaging advances which have allowed improved diagnosis as well as better monitoring of treatment response in IIMs. These imaging techniques complement currently available outcome measures and may play a critically important role in future clinical trials.

MRI is the most studied imaging technology in IIMs. It may be used to support a clinically suspected diagnosis when other standard measures are not helpful. Once validated, it can possibly be used longitudinally to assess disease activity and muscle damage more rigorously. While initially only the T1 and T2 weighted sequences were used for assessment, more recent advances have allowed for the development of newer sequences that provide additional information, and there is potential to capture the early stages of myositis prior to the development of lab abnormalities. MRI is sensitive in showing muscle edema and active myositis (16, 17, 28). Quantitative techniques, such as Dixon fat-water imaging (quantifies tissue fat on a scale of 0%−100%) can be more precise and reliable than traditional visual radiographic methods.

Arguments against the use of MR imaging include the fact that it is expensive, may take a long time to complete, and requires prolonged immobility in potentially uncomfortable positions. Over the past few decades, the cost of obtaining MR imaging has decreased significantly, and the availability of MR is more widespread. Additionally, new techniques are offering faster imaging while preserving quality. However, at the present state, MRI may not be sufficient enough to make a diagnosis of IIM. While MRI captures muscle pathology, particularly muscle edema and fat infiltration, as mentioned earlier, none of these changes are specific for muscle inflammation, and similar findings can also be seen in some muscular dystrophies and hereditary myopathies such as Pompe disease (165–167). Today, the most common uses of MR imaging are to help target biopsy sites and to possibly aid in diagnosis in difficult cases. MR imaging has the potential to enable clinicians and researchers to better track disease management and progression. However, the required frequency of MRI images to adequately capture disease progression or the time interval necessary to satisfactorily determine the impact of an intervention remains undetermined. Similarly, whole-body MRI is becoming popular, particularly in the pediatric age group, but whether whole-body MRI has a diagnostic or prognostic advantage over standard MRIs of specific muscles (for example, commonly employed thigh muscles) is yet to be answered. Furthermore, standardization of methods for research use remains a major challenge.

Another tool that is becoming an important part of the myositis specialist arsenal is muscle ultrasound (115). Neuromuscular ultrasound is non-invasive, easy to use, and rapid. It is roughly 80% sensitive in confirming muscle involvement and supporting the clinical diagnosis of an IIM. Neuromuscular ultrasound allows for rapid screening of a good number of muscles in suspected IIM cases in addition to needle electromyography during clinic visits though comparative studies are not available. Additionally, ultrasound can identify abnormal muscle morphology making it well-suited for use in targeting muscle biopsy and possibly enhancing its diagnostic yield. It is also more cost-effective than MR imaging.

Many other technologies, including EIM and PET, have shown promise in the imaging of muscle tissues, and studies are ongoing. Eventually, some of these new technologies will be integrated into the management and tracking of patients with IIMs after further validation as disease biomarkers. We believe that these imaging modalities may result in a paradigm shift in future in the diagnosis and management of the IIMs. Optimized imaging technologies may add another dimension to the tracking of treatment response during the treatment of IIMs. The use of these new technologies may lead to the design of more efficient screening clinical trials with smaller sample sizes and shorter duration which will be invaluable for the treatment of rare diseases like the IIMs.

MRI is the most widely studied imaging technology in IIM, and despite its limitation, it has major advantages over US or EIM (Table 1). Only limited studies have examined the use of US or EIM in myositis, and more data are warranted to define their role in the management of myositis. Both US and EIM are easy to use and cost-effective. They can be regularly employed in the office setting to track disease progression and may also provide complementary information to MRI findings. However, none of them are self-sufficient biomarkers of IIM. A head-to-head comparison study between the imaging technologies is lacking is much warranted.

## Conclusion

There have been significant advances in imaging technologies in the past two decades that have impacted the clinical practice and management of patients with IIMs. These evolving technologies have the potential to provide an objective assessment of muscle health once their utility is confirmed in large-scale prospective studies. Eventually, we anticipate that these technologies will be incorporated into a validated paraclinical assessment system that allows for more sensitive tracking of disease activity and treatment response to therapeutic interventions both in the clinic and in the context of therapeutic clinical studies.

## Author contributions

AZ reviewed all the abstracts from the initial search results and identified the articles. SS provided some of the imaging used in these manuscripts and contributed to the MRI section of the manuscript writing. MD critically reviewed this manuscript and also updated several sections of the manuscript and developed the table used in this manuscript. BR designed and developed the original draft along with AZ. PM critically reviewed the manuscript and added some sections on MRI imaging and updated the manuscript as needed. All authors contributed to the article and approved the submitted version.

## Supplementary material

Supplementary Figure 1 Literature review and search strategy.
