# Supplementary figures and images for: Imaging biomarkers in the idiopathic inflammatory myopathies
Source: Front Neurol. 2023 Apr 25;14:1146015. doi: 10.3389/fneur.2023.1146015 (PMC10166883; doi:10.3389/fneur.2023.1146015)

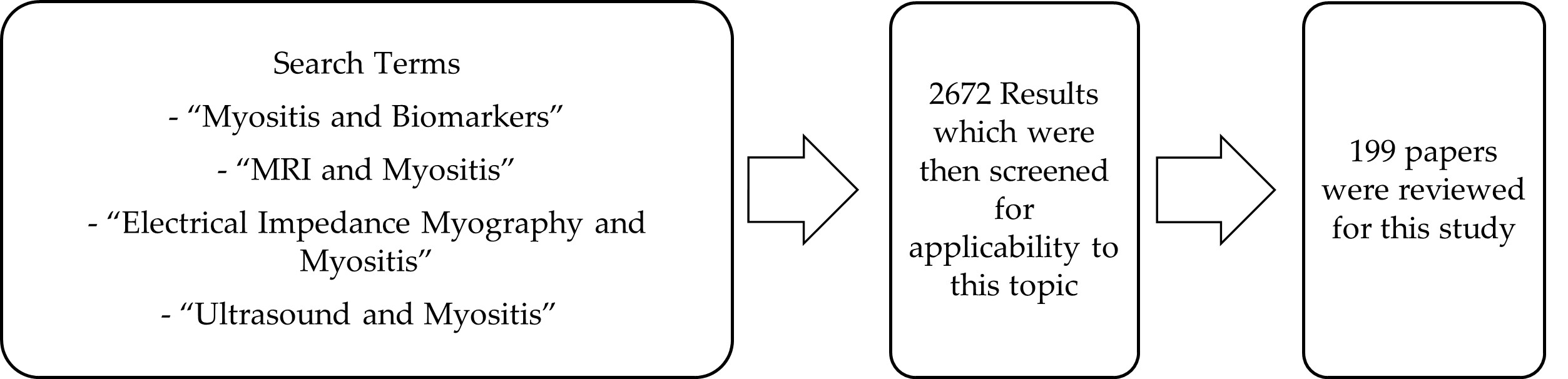

Supplement: Supplementary Figure 1 — Literature review and search strategy. [file Image_1.jpeg]
